# *Β*-Amylase from Starchless Seeds of *Trigonella Foenum-Graecum* and Its Localization in Germinating Seeds

**DOI:** 10.1371/journal.pone.0088697

**Published:** 2014-02-14

**Authors:** Garima Srivastava, Arvind M. Kayastha

**Affiliations:** School of Biotechnology, Faculty of Science, Banaras Hindu University, Varanasi, India; University of Insubria, Italy

## Abstract

Fenugreek (*Trigonella foenum-graecum*) seeds do not contain starch as carbohydrate reserve. Synthesis of starch is initiated after germination. A *β*-amylase from ungerminated fenugreek seeds was purified to apparent electrophoretic homogeneity. The enzyme was purified 210 fold with specific activity of 732.59 units/mg. *M_r_* of the denatured enzyme as determined from SDS-PAGE was 58 kD while that of native enzyme calculated from size exclusion chromatography was 56 kD. Furthermore, its identity was confirmed to be β-amylase from MALDI-TOF analysis. The optimum pH and temperature was found to be 5.0 and 50°C, respectively. Starch was hydrolyzed at highest rate and enzyme showed a *K_m_* of 1.58 mg/mL with it. Antibodies against purified Fenugreek *β*-amylase were generated in rabbits. These antibodies were used for localization of enzyme in the cotyledon during different stages of germination using fluorescence and confocal microscopy. Fenugreek *β*-amylase was found to be the major starch degrading enzyme depending on the high amount of enzyme present as compared to *α*-amylase and also its localization at the periphery of amyloplasts. A new finding in terms of its association with protophloem was observed. Thus, this enzyme appears to be important for germination of seeds.

## Introduction

*β*-Amylase (E.C. 3.2.1.2), member of family 14 of glycosyl hydrolases, catalyses the release of successive *β*-maltose from the non-reducing ends of *α*-1,4 linked oligo and poly glucans. The enzyme is distributed in higher plants and some micro-organism. *β*-Amylase is major contributor of diastatic power (i.e. the combined *α*-amylase, *β*-amylase, debranching enzyme and *α*-glucosidase activities) of malt. Exclusive maltose production is utilised in pharmaceutical industry for dispensing, production of maltose rich syrups, and non-digestible sweetner, maltitol [1].

The enzyme is synthesised and accumulated during grain development [2]. In germination of cereal seeds, *β*-amylase is known to play a vital role, where it is present in free and bound forms [1]. *De-novo* synthesis of the enzyme is reported during early germination of rice [3]. *β*-Amylase has also been shown to play a more important role as compared to *α*-amylase during early hours of germination in wheat scutella [4].

In plants, *β*-amylase is known to play primary role in hydrolysis of starch. The identification of *β*-maltose as a predominant sugar exported from the chloroplast at night, points towards role of *β*-amylase in degradation of transient starch [5]. Furthermore, study of transgenic and mutant plants lacking enzyme also showed its importance in starch hydrolysis [6]. Its association with starch granules was shown in endosperm of rice [7]; also the enzyme was found in the starchy endosperm of barley [8]. Abiotic stresses are also known to affect the activity and expression of *β*-amylases [9], [10], [11]. During stress, stimulation of starch degradation takes place in leaves for supporting respiration under condition of low photosynthesis and to protect cell structure by raising the levels of maltose as an osmoprotectant [11].

But, this role cannot be extended to all plant *β*-amylase, where it is known to perform various physiological roles. Its role as a storage protein was suggested in case of taproots of alfalfa (*Medicago sativa*) [12], cereal endosperm [1] and hedge bindweed rhizome (*Calystegia sepium*) [13]. In barley seeds *β*-amylase may have a nitrogen-storage function, since the profile of accumulation of *β*-amylase in developing seed resembles to that of the storage protein, hordein, and the synthesis of *β*-amylase responds to increased supply of nitrogen [14].

The extra chloroplastic localization of enzyme in spinach cells [15] and *Arabidopsis* leaves [16], provides evidence for its non-amylolytic function. Nine genes of *Arabidopsis* are known to encode for *β*-amylase isozymes. Six members of the family are predicted to be extrachloroplastic isozymes and three contain plastid transit peptides [17]. In leaves of pea (*Pisum sativum*) and wheat (*Triticum aestivum*) [18], the enzyme was found to be present in vacuole, where the assessment of its role is difficult, as the identification of vacuolar substrate remains to be determined. Furthermore, studies of proper germination of *β*-amylase deficient lines of soybean (*Glycine max*) [19] and rye (*Secale cereal*) [20] demonstrated its non-essential role in carbohydrate metabolism. *In vitro* studies have shown that native starch granules are not degraded by β-amylase without prior digested by other enzymes, making its role unclear [21]. The localization of enzyme was also found in phloem, by raising monoclonal antibody against sieve elements of tissue culture of *Streptanthus tortuosus* into BALB/c mice. The antibody identified enzyme to be present in phloem forming tissue cultures but absent in cultures lacking phloem. This antibody also cross-reacted with the major form of *A. thaliana β*-amylase and showed binding with its sieve elements [22].

Fenugreek (*Trigonella foenum-graecum* L. Leguminosae) is one of the oldest medicinal plant originating in India and North Africa. It has a long history of medicinal uses in Ayurvedic and Chinese medicine. It has been used for labour induction, aiding digestion and for improving metabolism and health. Apart from its use as a medicinal plant it is commonly used in cuisine. The seed contains many nutrients including protein, carbohydrates, fat in the form of volatile and fixed oil, vitamin and minerals as well as enzymes, fiber, saponins, choline and trigonelline [23]. During fenugreek seed germination and seedling development three different types of carbohydrates (galactomannans, soluble sugars including galactosyl sucrose sugars and starch) are utilized [24]. Fenugreek seeds are starchless; galactomannans being the main carbohydrate reserve. Synthesis of starch starts only after germination [25]. Cotyledon was found to be the major site of starch accumulation [24]. Thus, making fenugreek seed an interesting material to study the potential role of *β*-amylase in carbohydrate mobilization, following germination.

Here, an attempt was made to study the physiological role of *β*-amylase in fenugreek seed and during seedling development, by purifying it from ungerminated seeds and characterizing it. Further, its immunolocalization studies were carried out in cotyledons till 62 h after germination.

## Materials and Methods

### Chemicals and Plant Materials

Fenugreek (*Trigonella foenum-graecum*) seeds were purchased from local market.

DEAE-cellulose, glycogen, amylopectin, maltose, pullulan, *α*-cyclodextrin, *β*-cyclodextrin, trypsin profile 1GD kit were purchased from Sigma Chemical Co., St. Loius; Mo, U.S.A.

Molecular markers for FPLC were from Pharmacia, Sweden.

All solutions were prepared in Milli Q (Millipore, Bedford, MA, U.S.A.) water.

All the chemicals for buffer were of analytical or electrophoresis grade from Merck Eurolab GmbH Darmstadt, Germany.

### Enzyme and Protein Assays

Enzyme activity was measured using Bernfeld’s method [26]. Reaction mixture was prepared by taking 0.5 mL of suitably diluted enzyme and 1% starch prepared in 50 mM sodium acetate buffer, pH 5.0. This was incubated at 30°C for 3 min, reaction was stopped by addition of 1 mL of 3,5-dinitrosalicylic acid. Test tubes were then placed in boiling water bath for 5 min and were allowed to cool down to room temperature, followed by addition of 10 mL of distilled water. Absorbance was recorded at 540 nm. One unit of *β*-amylase is defined as the amount required for release of 1 *µ*M of *β*-maltose per min at 30°C and pH 5.0 under the specified condition.

For measurement of α-amylase activity, the extract was heated at 70°C for 15 min and assayed as described above. The activity was also checked using starch azure as substrate.

Amount of protein present in sample was determined by Folin’s method using crystalline BSA as standard protein. The protein profiles in column chromatography were followed by measuring the absorbance of the eluates at 280 nm.

### Enzyme Purification

All the steps were performed at 4°C and centrifugation was carried out at 8,720 *g*, unless stated otherwise.

50 g of fenugreek seeds were soaked in 50 mM Tris-HCl buffer, pH 7.2, for 12 h. Seeds were coarsely crushed using waring blender in extraction buffer (50 mM Tris-HCl buffer, pH 7.2 containing 1 mM EDTA and 1 mM PMSF), and then filtered through two layers of pre-washed muslin cloth. The extract thus prepared was centrifuged for 20 min.

Crude extract was subjected to 50–65% acetone fractionation at −15°C. The precipitated proteins obtained after centrifuging it for 20 min were made free of acetone and then dissolved in extraction buffer. Solubilized proteins were loaded onto DEAE-cellulose column equilibrated with 25 mM Tris-HCl buffer, pH7.0. Enzyme was eluted and 2 mL fractions were collected with an increasing gradient of 0–0.2 M NaCl prepared in the same buffer, with a flow rate of 0.3 mL/min. The enzyme active fractions were pooled and dialysed against extraction buffer.

The dialyzed sample was used for affinity precipitation by following the method as described by Silvanovich and Hill [27] with some modifications. The sample was saturated with ethanol at −15°C. Precipitated and centrifuged for 20 min, the proteins precipitated were discarded. Glycogen solution (2%) was added dropwise to the supernatant with stirring at 4°C for 20 min followed by centrifugation for 15 min. The pellet thus obtained was washed twice with extraction buffer containing 40% ethanol. The washed pellet was suspended in 1 mL extraction buffer and kept at 37°C for 1 h for hydrolysis of glycogen and dialyzed it against extraction buffer. After dialysis it was centrifuged for 15 min. The supernatant was collected and small molecular oligosaccharides were removed by dialyzing against extraction buffer, overnight.

### Electrophoresis

SDS-PAGE was used to check the homogeneity of each fraction. Analytical SDS-PAGE of 12% polyacrylamide gel was performed according to Laemmli’s method [28], using vertical gel electrophoresis apparatus (Monokin, India). Proteins were visualized by staining with Coomassie Brilliant Blue R-250 (CBB R-250). The apparent molecular mass of *β*-amylase was determined by comparing with relative mobility of protein standards (molecular mass ranging from 72 kD to 290 kD). For, detection of enzyme activity, Native-PAGE (8%) was carried at 4°C in the same manner as SDS-PAGE but in the absence of SDS. Activity staining was performed by incubating gel at 30°C in 1% starch prepared in 0.1 M sodium-acetate buffer, pH 5.4, for 15 min followed by staining with 0.01 N I_2_-KI solution.

To determine the pI of β-amylase, the glycogen precipitated sample was subjected to Isoelectric Focussing (IEF) with an Ettan IPG phor 3 instrument (GE Healthcare Lifesciences Ltd., Uppsala, Sweden) using an 11 cm IPG strip (pH range 3–10) by following protocol described elsewhere [29]. Protein was visualized using CBB R-250.

### Native Molecular Mass Determination

Size-exclusion chromatography using Sephacryl S-200 (Hiprep 16/60 High resolution column, GE Healthcare Lifesciences Ltd., Uppsala, Sweden) on AKTA FPLC was performed for determination of native molecular weight of purified protein. The column was pre-equilibrated with 50 mM sodium acetate pH 5.0 containing 0.1 M NaCl. The column was calibrated with five proteins in molecular range of (12.5–240 kD). The enzyme was eluted with the same buffer at a flow rate of 0.5 mL/min. Absorbance was recorded at 280 nm and fractions corresponding to peak were checked for enzyme activity as described above. The native molecular mass was determined from a plot of Ve/Vo against log of molecular mass of standard proteins taken.

### Glycoprotein Properties

In order to ascertain whether enzyme is glycoprotein, 0.2 mL of purified *β*-amylase was taken in a test tube and to this 0.1 mL of 10 mM sodium metaperiodate was added and incubated at 30°C for 10 min. Exposure to air was avoided and 0.3 mL of 0.5% Schiff’s reagent was added to the test tube and incubated at 30°C for 1 h, absorbance was recorded at 550 nm.

### MALDI-TOF Mass Spectrometry

The identification and molecular weight determination of the protein band obtained after SDS-PAGE was performed using Matrix Assisted Laser Desorption Mass Spectrometry (MALDI; Voyager-DE-STR instrument; Applied Biosystems, USA). The protein band was excised from SDS-PAGE gel, destained, washed and digested with MALDI grade trypsin (Sigma, St. Louis, USA) overnight, following the protocol described by Kishore et al. [30]. The peptides obtained were analysed by matching with the sequence available in database. The amino acid sequence of *β*-amylase from *Trigonella foenum-graecum* is not available in protein database. Therefore, the peptide mass fingerprint obtained after MALDI-TOF was for matched with *β*-amylase from other plant sources using MASCOT (www.matrixscience.com) and applying following parameters: (a) fixed modifications carbamidomethyl (C); (b) variable modifications oxidation (M); (c) cleavage by trypsin: cuts C-term side of KR unless next residue is P.

### Substrate Specificity

The ability of amylase to degrade various polysaccharides was determined by performing assay using 1% solution of starch (insolubilized and solubilized), amylopectin, glycogen, pullulan, and starch azure. For performing the assay using chromogenic substrate, starch azure, following procedure was followed: 4.5 mL of 2% starch azure suspension was prepared in 0.02 M sodium phosphate buffer, pH 7.0, containing 0.05 M NaCl and equilibrated at 37°C, followed by addition of 0.5 mL of suitably diluted enzyme. It was mixed by swirling and incubated at 37°C for 15 min in a water bath. Reaction was stopped by addition of 20 mL of 2.75 M acetic acid. The suspension was filtered through Whatman filter paper no. 2 and its absorbance was recorded at 595 nm [31].

### Effect of EDTA

The amylase was dialyzed overnight against buffer containing 5 mM EDTA, followed by determination of the residual activity under standard condition.

### Paper Chromatography

Identification of low molecular weight products of starch hydrolysis formed due to enzyme action, was performed by paper chromatography using butanol:pyridine:water (6∶4: 3; v/v) solvent system. 5% starch solution was incubated with enzyme overnight, which was then kept in boiling water bath for 5 min to denature the enzyme, and was removed by centrifugation for 20 min. The supernatant was loaded onto the Whatman paper and chromatograms were developed for 4 h. Reducing sugars were visualized by treating it with a solution of 0.5% 3,5-dinitrosalicylic acid reagent and 4% NaOH [32], [33].

### Kinetic Studies

Enzyme obtained after glycogen precipitation was used for the kinetic studies. Effect of pH was observed by using the following buffers 0.05 M sodium-acetate buffer (pH range 3.6 to 5.6), 0.05 M phosphate buffer (pH range 6.0 to 7.0), 0.05 M Tris-HCl buffer (pH range 7.2 to 9.0). Enzyme activity was assayed by preparing starch solution in the appropriate buffers and following the procedure described earlier. Optimum temperature was determined by carrying out assay procedure in temperature range 20 to 70 (±1)°C, and activation energy (*E_a_*) for *β*-amylase was calculated from the slope of the curve using an Arrhenius plot in the range of 20 to 50 (±1)°C. Thermal inactivation study for the enzyme was performed by maintaining enzyme at 52°C in a water bath (Multitemp, Pharmacia, Sweden). Small aliquots withdrawn at different time intervals were assayed for enzyme activity. Effect of substrate concentration was observed by using starch and amylopectin in the range of 0–10 mg/mL (pH 5.0), data thus obtained was used for determination of Michaelis constant (*K_m_*) and maximum velocity (*V_max_*) using Lineweaver-Burk plot. Enzyme was incubated for 10 min at 30°C with Schardinger dextrins, sucrose and maltose, so as to observe their effect on enzyme activity (for determining effect of maltose on enzyme, assay was carried out according to Fuwa’s method [34]). Inhibition constant (*K_i_*) for *α*-cyclodextrin and sucrose was determined using Dixon plot. All parameters were the mean of triplicate determinations from three independent preparations.

### Preparation of Specific Antibodies against β-amylase

Antibody against Fenugreek *β*-amylase was raised by IMGENEX, Bhubneshwar, India; by injecting purified protein into rabbits, consequently the polyclonal antiserum was developed and antibody generated was purified using Protein-A affinity chromatography. These antibodies were used for blotting analysis.

### Seed Germination

50 g of dry seeds were surface sterilised with 0.5% hydrogen peroxide, washed four times with water and left in water to imbibe for 12 h at 30°C. The seeds which had swollen were set out to germinate over moist filter paper on moist sand bed.

### Western Blot Analysis

Protein (30 *µ*g) from crude extract was denatured and resolved by 10% SDS-PAGE and transferred onto PVDF membrane (Sigma, St. Louis,USA). Transfer efficiency was checked by Ponceau-S staining, which was later completely removed from the membrane by repeated washing in water. The membrane was blocked with 5% non-fat milk in Phosphate Buffer Saline (PBS) overnight at room temperature, followed by incubation with anti-*β*-amylase polyclonal antibody raised in rabbit (1∶1000) for 6 h. Subsequently, the blot was washed two times (5 min each) in PBS-0.1% Tween 20 and incubated with goat anti-rabbit IgG-horse-radish peroxidase conjugate (1∶2000) (Banglore Genei, India) for 3 h at room temperature. The blot was washed thrice (5 min each) in PBS-0.1% Tween 20 and detected by enhanced chemiluminescence (ECL).

### Immunohistochemistry

Slices (2 mm) of cotyledon tissue were fixed in 3% glutaraldehyde in 0.2 M phosphate buffer (pH 7.2), dehydrated and embedded in paraffin wax. For light and fluorescence microscopy, sections were prepared using Leica RM2245 semi-motorized rotary microtome. Approximately 6 *µ*m thick sections were cut and collected on polylysine coated slides.

Prior to staining sections were deparaffinised using xylene and rehydrated using 100%, 90%, 70%, 50% and 30% alcohol, respectively in sequential order, for 5 min each.

Sections were pre-incubated in 5% (w/v) BSA in PBS at pH 7.4 for 30 min, then incubated for 4 h in primary antibody diluted in PBS containing 1% BSA and 0.32% Tween 20 at 1∶500 dilution. Slides were rinsed four times for 15 min with PBS-Tween, then incubated for 2 h, in the dark with secondary antibody (Goat Anti-rabbit IgG (H+L)-FITC conjugate) diluted 1∶300 in PBS, 1% BSA, 0.32% Tween 20. Slides were then rinsed 4 times with PBS- Tween for 15 min each [35]. Longitudinal sections were analysed using fluroscence microscope (Nikon DS-Fi1) or Laser Scanning Confocal Microscope (Nikon Eclipse Ti microscope) at 488 nm excitation wavelength for fluorescein isothiocyanate (FITC). Images were processed using the software MetaMorph and CorelDRAW Graphics Suite X6.

### Photomicroscopy

Sections approximately 6 *µ*m thick were cut and collected on polylysine coated slides. Staining of section with 0.1% (w/v) toluidine blue in 1% (w/v) borax, pH 11 was done for general structure observations [35].

To determine the location of starch deposits in cotyledons of ungerminated and germinated seeds, semithin sections of seeds were stained with I_2_-KI solution [25]. Slides were observed under light microscope (Nikon DS-Fi1).

## Results

### Purification

**Figure 1:**
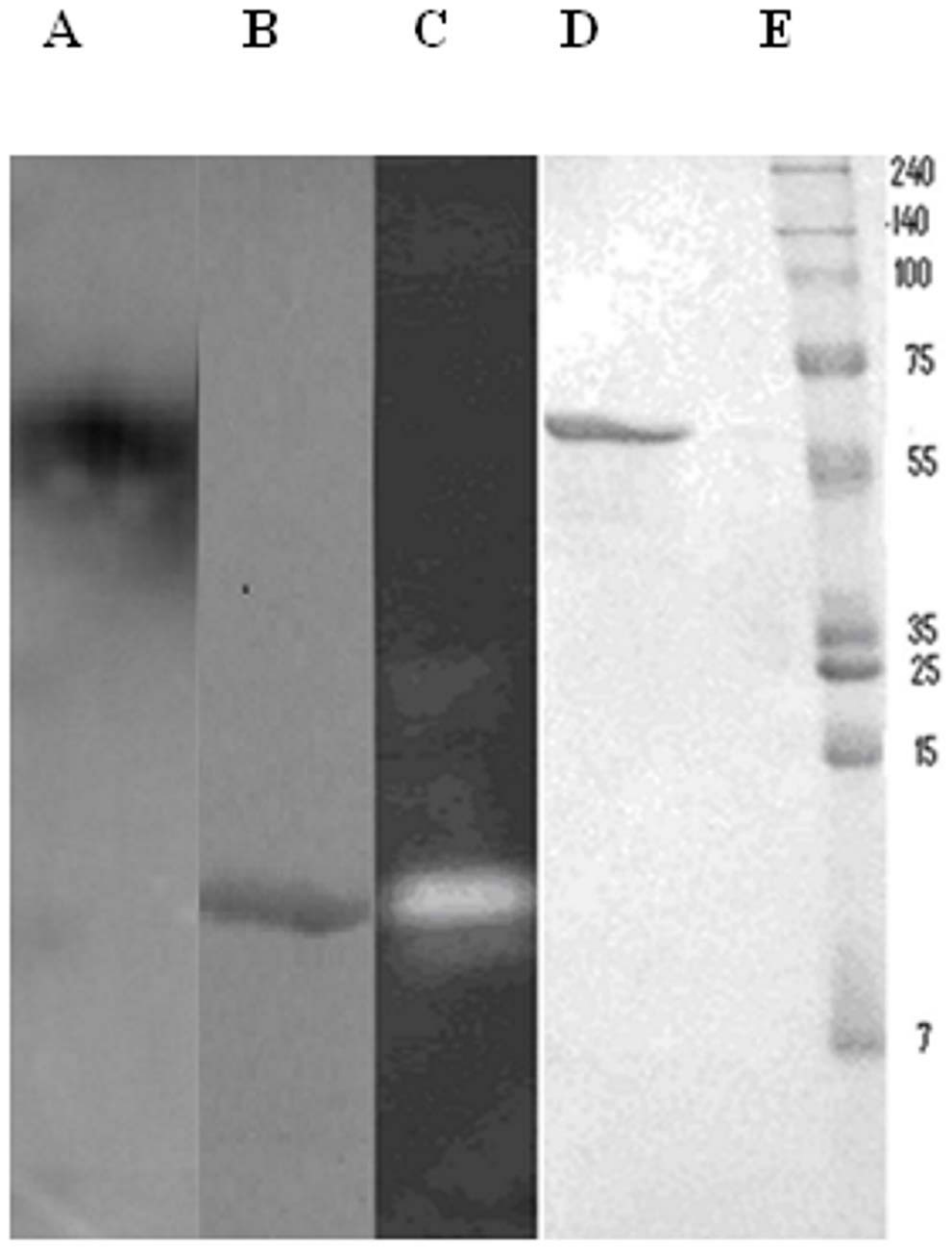
Electrophoresis pattern of Fenugreek *β*-amylase. Lane A : Western blotting of *β*-amylase, B: Native PAGE, C:Activity staining, D: SDS-PAGE of purified *β*-amylase, E: Molecular weight markers.

Crude extract was prepared from ungerminated seeds, as described under experimental section. The fractionation of proteins by acetone followed by anion-exchange chromatography using DEAE-cellulose and glycogen precipitation, as outlined in table 1, resulted in purification of Fenugreek *β*-amylase 210 fold with a yield of 21%. Homogeneity of the sample obtained after glycogen precipitation was checked on SDS-PAGE (Fig. 1 D), which revealed a single band after CBB staining. *M_r_* of enzyme was determined to be 58 kD, when compared to standard molecular weight markers. Activity staining showed development of clear band (site of activity of *β*-amylase) over a dark background formed due to complex of starch and I_2_-KI solution (Fig. 1 C), corresponding protein band on Native-PAGE is shown in Fig. 1 B. The purified protein gives a single peak from Sephacryl S-200 gel filtration column and the molecular mass of native Fenugreek *β*-amylase determined from the mean of two experiments with it was 56 kD, which is in accordance with the mass obtained on SDS-PAGE, hence suggesting monomeric nature of enzyme. pI of Fenugreek *β*-amylase was determined to be 5.2 from IEF. The peaks obtained after MALDI-TOF analysis and the peptide mass fingerprints were used for database searching using MASCOT. The most significant match was found with *β*-amylase of *Medicago sativa* (alfalfa; Accession no. T09300) with a score of 82 and E-value of 0.0015 (Table 2).

**Table 1:** Purification of *β*-amylase from *Trigonella foenum-graecum* (fenugreek) (50 g).

Steps	Total Activity(units)	Total Protein(mg)	Specific Activity(units/mg)	Purification (fold)	Recovery (%)
**Crude**	2845.34	769.8	3.49	_	_
**Acetone fractionation (50–65%)**	2273.87	75.8	29.59	8.28	79.8
**Ion Exchange (DEAE-Cellulose)**	1365.76	6.8	200.85	57.54	48
**Glycogen precipitation**	586.07	0.8	732.59	209.91	20.6

Fold purification calculated with respect to the specific activity of the crude extract.

**Table 2:** Sorted peptide according to their residue number and masses along with peptide match.

Start-End	Observed	Mr(expt)	Mr(calc)	Delta	Miss	Sequence
150–160	1333.5499	1332.5426	1332.6271	−0.0845	0	R.TAIEIYSDYMK.S
161–171	1339.5571	1338.5498	1338.6601	−0.1103	1	K.SFRENMSDLLK.S
324–331	919.4445	918.4372	918.4923	−0.0551	0	R.DGYRPIAK.I
324–331	919.4445	918.4372	918.4923	−0.0551	0	R.DGYRPIAK.I
336–348	1641.6852	1640.6779	1640.7915	−0.1136	0	R.HHAILNFTCLEMR.D
336–348	1657.6980	1656.6907	1656.7864	–0.0957	0	R.HHAILNFTCLEMR.D
374–386	1401.6140	1400.6067	1400.6895	−0.0828	0	R.ENIEVAGENALSR.Y
387–406	2249.0198	2248.0125	2248.1600	−0.1474	0	R.YDATAYNQIILNARPQGVNK
414–421	1002.4468	1001.4395	1001.5004	−0.0609	0	R.MYGVTYLR.L
414–421	1002.4468	1001.4395	1001.5004	−0.0609	0	R.MYGVTYLR.L
414–421	1018.4418	1017.4345	1017.4953	−0.0608	0	R.MYGVTYLR.L
414–421	1018.4418	1017.4345	1017.4953	−0.0608	0	R.MYGVTYLR.L

### Characterization of Fenugreek β-amylase

Enzyme hydrolyzed starch at highest rate followed by amylopectin, amylose and glycogen. Pullulan was not hydrolyzed indicating that enzyme failed to cleave *α*-1,6 bond. Also, the inability to give colour with assay performed using starch azure as substrate suggested that it was endoamylase. Fenugreek *β*-amylase was not able to degrade native starch (Table 3). There was no loss in enzyme activity after dialysis against buffer containing 5 mM EDTA for overnight, which indicates that Fenugreek *β*-amylase is not a metallo-enzyme. The characteristic property of *β*-amylase to release exclusively maltose as end product of starch hydrolysis was identified by paper chromatography. Periodic acid-Schiff’s reagent method showed absence of any carbohydrate in the purified enzyme, indicating its non-glycosylated nature. The effect of pH on purified *β*-amylase was examined in the pH range 3.6 to 8.0. Maximum activity was observed at pH 5.0 in 50 mM sodium acetate buffer and enzyme was found to be fairly stable for a week in pH range of 3.0–7.5. Optimum temperature was found to be 50°C. The enzyme activity showed decline on increasing temperature beyond 50°C; complete loss of enzyme activity was observed when it was kept at 70°C for 15 min. The value of activation energy (*E_a_*) was calculated to be 6.21 kcal/mole from Arrhenius plot. The *K_m_* value for starch and amylopectin as a substrate was found to be 1.58 mg/mL and 2.86 mg/mL, respectively. The highest specific activity of *β*-amylase gave a turnover number of 137.93 min^−1^.Thermal inactivation studies were carried out at 52°C, and it was found to follow first-order kinetics with rate constant of 0.0198 min^−1^ and t_½_ equal to 35 min. When enzyme was incubated with 1 mM of Schardinger dextrins; *α*-cyclodextrin showed inhibitory effect and was found to be a competitive inhibitor from Dixon plot method with *K_i_* 3.97 mM. Sucrose was also found to be a competitive inhibitor of Fenugreek *β*-amylase with *K_i_* 2.32 mM, while maltose did not show any inhibitory effect on enzyme activity even at a concentration of 25 mM.

**Table 3:** The effect of various substrates on the enzyme activity.

S. No.	Substrate	Relative Activity (%)
**1**	Starch (solubilized)	100.00
**2**	Amylopectin	99.12
**3**	Amylose	50.30
**4**	Glycogen	43.00
**5**	Pullulan	0.04
**6**	Starch (insolubilized)	0.00

### Localization of the β-amylase in the Cotyledons of Fenugreek

Fenugreek seeds were soaked in water for 12 h and then germinated on sand bed for different time intervals. Radicles were cleaved from the cotyledons and were fixed in 3% glutaraldehyde, further they were embedded in paraffin wax followed by sectioning. Sections were collected on polylysine coated slides. Toluidine blue staining was done for observing general histology of cotyledons after 14, 31 and 62 h of germination. Comparable samples of seed were stained with I_2_-KI solution for detection of starch. To investigate the cellular localization of *β*-amylase during ungerminated and germination stages, immunohistochemistry was performed. The sections obtained from paraffinized blocks were immune-reacted with the purified antibodies raised against Fenugreek *β*-amylase. Comparable samples were also treated in the same manner with non-immunized rabbit serum as control. The specificity of the affinity purified antibodies was checked by western blot analysis of crude extract of seed. Antibodies specifically reacted with the 56 kD polypeptide corresponding to the Fenugreek *β*-amylase (Fig. 1 A).

**Figure 2:**
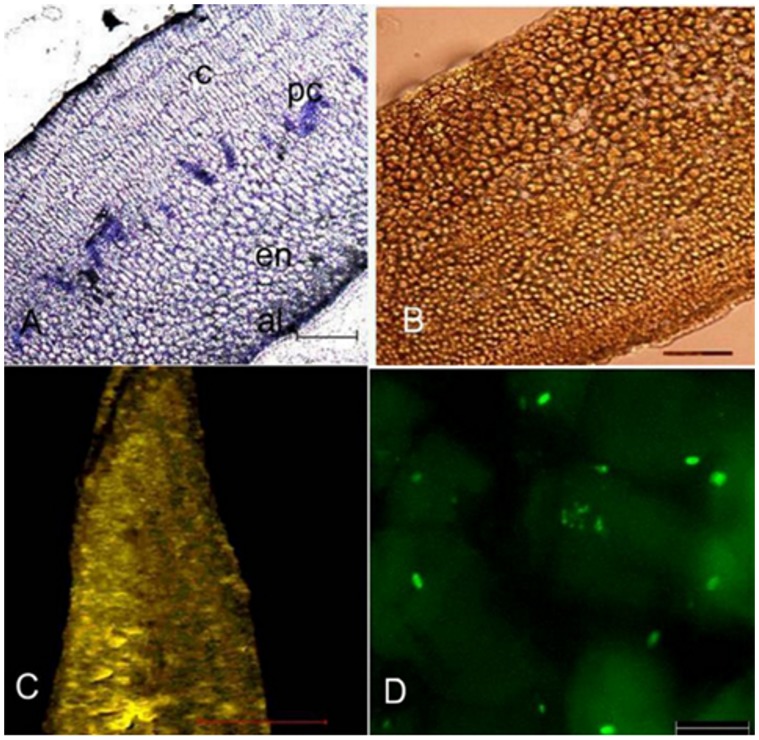
General histology of fenugreek seed and immunohistochemical localization of Fenugreek β-amylase. Thin sections of seeds of *Trigonella foenum-graecum* (A) stained with toluidine blue for observing general histology (B) stained with I_2_-KI solution for visualization of starch (C) section labelled with anti-(*β*-amylase) IgG showed localization of enzyme in the endosperm (D) enlargement of (C) showing part of the endosperm. Bars represent 100 *µ*m in A and B, 200 *µ*m in C and 20 *µ*m in D. cotyledon cells (*c*), procambial cells (*pc*), endosperm cells (*en*), aleurone cells (*al*).

**Figure 3:**
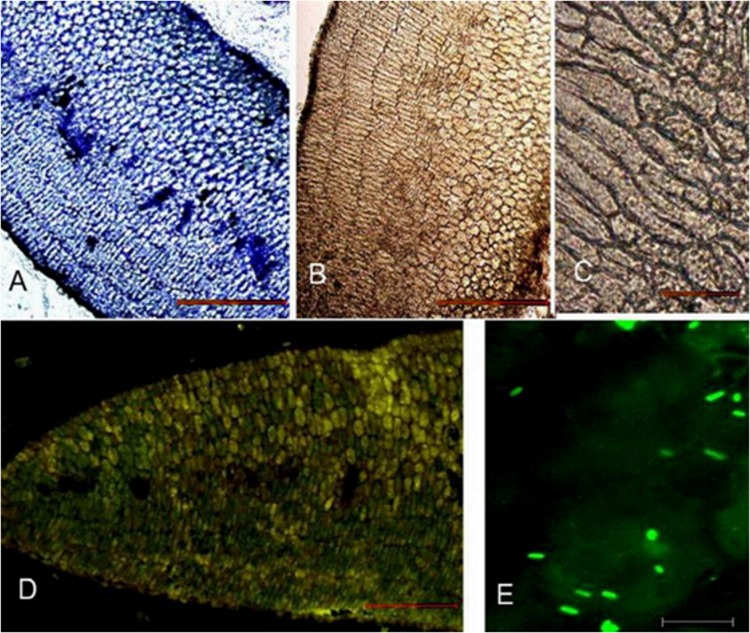
Thin longitudinal sections of cotyledons of *Trigonella foenum-graecum* after 14 h germination. (A) Toluidine blue stained section (B) Section stained with I_2_-KI solution showed presence of starch in few cotyledon cells (C) Detail of (B) showing part of cotyledon cells (D) Immunolocalization shows presence of enzyme in endosperm and few cotyledon cells (E) Enlargement of (D) showing endosperm cells. Bars represent 120 µm in A, B and D, 45 *µ*m in C and 20 *µ*m in E.

**Figure 4:**
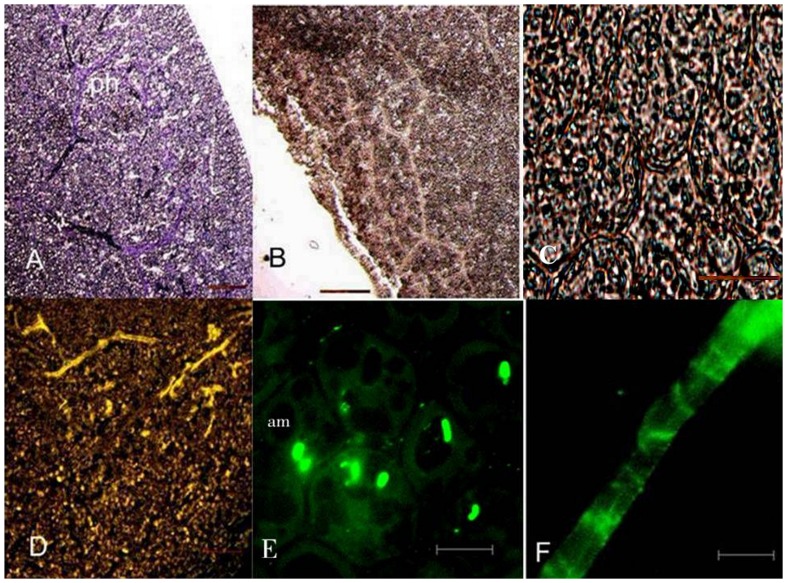
Longitudinal sections of cotyledon of *Trigonella foenum-graecum* after 31 h germination. (A) Toluidine blue stained section (B) I_2_-KI solution stained section (C) Detail of (B) showing amyloplasts (D) Immunolabelling of *β*-amylase by using anti-(*β*-amylase) (E) Enlargement of (D) shows presence of enzyme associated with amyloplasts and in (F) with protophloem. Bars represent 100 *µ*m in A, B and D, 45 *µ*m in C and 20 *µ*m in E and F. phloem (*ph*), amyloplasts (*am*). No fluorescence was seen in the sections concomitant to the sections stained when incubated with FITC-conjugated secondary antibody without prior treatment with primary antibody.

**Figure 5:**
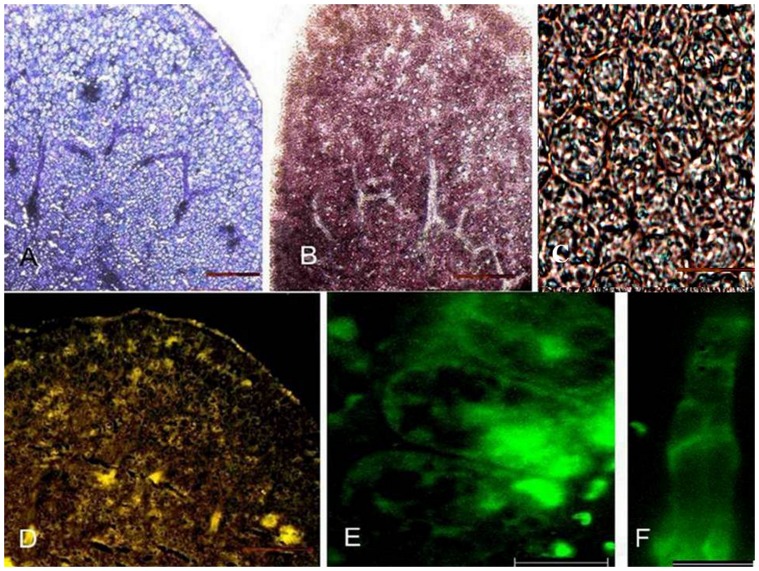
Longitudinal sections of cotyledons of *Trigonella foenum-graecum* after 62 h germination. (A) Sections stained with toluidine blue for general histology visualization (B) I_2_-KI stained sections show distribution of starch (C) Detail of (B) showing amyloplasts (D) Immunolocalization of *β*-amylase (E) Enlargement of (D) shows association with amyloplasts and in (F) with proto-phloem. Bars represent 100 *µ*m in A, B and D, 45 *µ*m in C and 20 *µ*m in E and F. Fluorescence was not observed in the sections treated with Goat Anti-rabbit IgG (H+L)-FITC conjugate antibodies without prior treatment with primary antibody.

The toluidine stained longitudinal section of fenugreek seeds showed one cell layer of aleurone cells surrounding the endosperm. The cotyledon cells were present below the endosperm (Fig. 2 A). The enzyme was found to be localised in the endosperm of ungerminated seeds (Fig. 2 C, D). Fluorescence was not observed in the cotyledonary cells and procambial cells. Comparable sections stained with I_2_-KI showed absence of starch (Fig. 2 B). Germination being defined as penetration of the seed coat by the radicle, occurred after about 10 h in the seeds set out for germination. No change in general histological structure of the seed was observed after 14 h of germination (Fig. 3 A). Starch could now be seen in cotyledonary cells (Fig. 3 B, C). *β*-Amylase was found to be present mainly in endosperm cells at this stage also, although some of the cotyledon cells also show fluorescence. Both amount of starch and β-amylase showed an increase at the stage of 31 h after germination. Toluidine blue stained sections showed dissolution of endosperm cell and the reserve stored (Fig. 4 A). Starch was predominantly present (Fig. 4 B, C). Fluorescence staining showed association of *β*-amylase with amyloplasts (Fig. 4 D, E). Enzyme also showed its association with protophloem at this stage of germination (Fig. 4 D, F). After 62 h *β*-amylase could still be seen in phloem and at the periphery of amyloplasts (Fig. 5 D, E, F). Although, at this stage decline in the amount of enzyme (Fig. 5 D) and starch was observed (Fig. 5 B, C).

**Figure 6:**
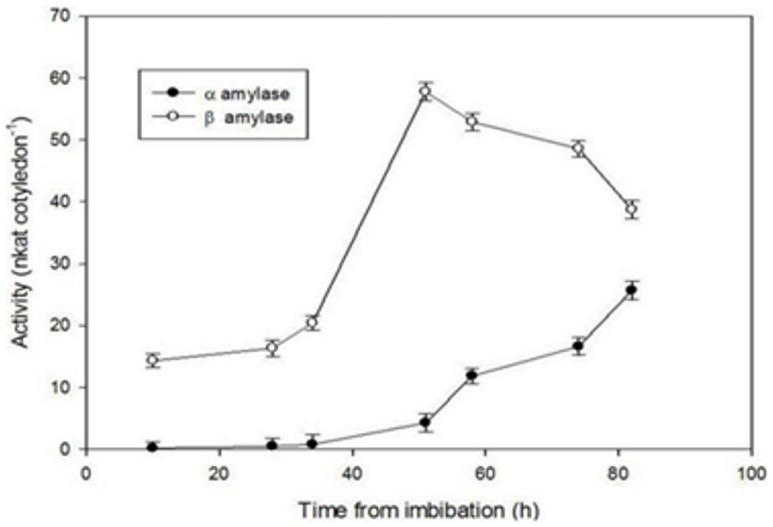
*α*- and *β*-amylase activity of crude extract of fenugreek from the seeds imbibed for upto 80 h.

The activity profile of *α*-amylase and *β*-amylase during germination of seed was observed by performing assay procedure as described above. *α*-Amylase activity was negligible up till 31 h and showed an increase after that, while *β*-amylase activity was comparatively quite higher and showed an increasing trend upto 41 h, thereafter decline was observed (Fig. 6).

## Discussion

The plant material used for purification of *β*-amylase consisted of ungerminated fenugreek seeds. Enzyme was purified by using steps as summarized in table. Affinity precipitation using glycogen proved to be a useful step for purification. *β*-Amylase from pea [36], maize [37] and radish [38] have also been purified using this method. Monomeric *β*-amylase are more commonly found in plants, having molecular mass in range of 42 to 65 kD [8], [33], [37], [38], *β*-amylase from sweet potato [39], leaves of *Vicia faba* [40] and hedge bindweed [13] are known to be tetramer having subunits of 64.7 kD, 26 kD and 56.07 kD, respectively. Fenugreek *β*-amylase was also found to be monomeric in nature. Fenugreek *β*-amylase resembled other plant *β*-amylase in terms of highest affinity for starch as compared to other substrates, pI, kinetic parameters and *α*-cyclodextrin being competitive inhibitor [41], [36], [13]. However, end product inhibition by maltose was not observed.

Fenugreek is a leguminous seed, having major carbohydrate reserve in the form of galactomannans stored in endosperm cells. During course of germination the hydrolysis of galactomannans take place and the break down products are absorbed by the cotyledons in which sugar increases and starch is formed [25], [42]. ADPGppase (adenosine diphosphate glucose pyrophosphorylase) is known to be a key regulatory enzyme for the formation of glycosyl donor for starch synthesis. Partial regulation of starch accumulation within cotyledons was suggested to occur by the transcription of ADPGppase genes, which shows positive correlation with the synthesis and decay of starch [24]. Temporary storage of starch takes place which gets remobilized during later time of seedling development [42]. *β*-Amylase was found to be present in the ungerminated seed, which did not contain starch. To study the probable role of *β*-amylase in fenugreek seeds, they were allowed to germinate for different time periods. In ungerminated seeds, enzyme was present in the endosperm cells. *β*-Amylase could be seen in the endosperm of seeds after 14 h of germination. Some of the cotyledon cells at this stage showed the presence of starch which were near endosperm cells. Thus, the substrate was inaccessible to the enzyme. The dissolution of endosperm cells was completed by 31 h and the cells showed presence of high amount of starch. *β*-Amylase content also showed an increase along with *α*-amylase (Fig. 6). *β*-Amylase could be seen at the periphery of amyloplasts. Similar to other plant *β*-amylases, Fenugreek *β*-amylase also showed inability towards degradation of the native starch granule. Therefore, presence of not high but significant amount of *α*-amylase may be required to initiate the reaction, followed by hydrolysis by *β*-amylase. At this stage of germination, starch acts as carbohydrate reserve and thus its hydrolysis is needed for germination and growth of seedling. The high amount of *β*-amylase present as compared to *α*-amylase and its presence at the periphery of amyloplasts defines the key role of *β*-amylase in hydrolysis of starch. Similar observations were made during germination of rice seeds [7] and barley seeds [43], where *β*-amylase was shown to be associated with starch granule. In sweet potato, cell walls and starch granules were labelled by the polyclonal antibodies raised against an inhibitor of starch phosphorylase, which was later identified as *β*-amylase [44]. After 62 h of germination amyloplasts were still surrounded by the enzyme. A decline in the content of starch and *β*-amylase was observed at this stage. The correlation between starch and *β*-amylase further confirms its role in starch hydrolysis.

The fluorescence could be seen in protophloem in the cotyledons after 31 h and 62 h of germination. Phloem is the primary transport for organic compounds in plants and cotyledons. The transported organic compounds serve as major substrate for plant growth. During the process of vascular differentiation, some of the provascular cells divide longitudinally to give rise to procambial cells, out of which some are destined to become phloem precursor cells [45]. Protophloem cells specified during embryogenesis differentiate with in the first three days of germination [46]. In *Arabidopsis* it has been shown that during embryogenesis phloem differentiation in the cotyledons occur earlier than in the axis [47]. Association of *β*-amylase with the protophloem after 31 h of germination can be related to its important role in transportation. There is an earlier report on presence of phloem specific *β*-amylase in *A. thaliana* and *Streptanthus tortuous* stems. It was suggested that the enzyme may have a role in prevention of starch build up during translocation of sugars in phloem sieve elements [22]. Probably, similar role can be attributed to Fenugreek *β*-amylase.

## Conclusion

The Fenugreek *β*-amylase was found to be major starch degrading enzyme of fenugreek based on localization studies. This study also lead to the new finding of association of *β*-amylase with the protophloem, which can further be explored for providing an insight into the phloem physiology.
